# Association Between Serum Vitamin D Levels and Periodontal Conditions According to Sleep Duration Using Data from Korea National Health and Nutrition Examination Survey

**DOI:** 10.3290/j.ohpd.b5795657

**Published:** 2024-10-24

**Authors:** Hyo-Jin Lee, Soo-Myoung Bae, Sun-Jung Shin, Bo-Mi Shin

**Affiliations:** a Professor, Department of Dental Hygiene, College of Dentistry; Research Institute of Oral Science; Research Institute of Dental Hygiene Science, Gangneung-Wonju National University, Gangneung, Republic of Korea. Conceptualisation, methodology, formal analysis, prepared original draft, reviewed and edited the manuscript, read and approved the final version of the manuscript.; b Professor, Department of Dental Hygiene, College of Dentistry; Research Institute of Oral Science; Research Institute of Dental Hygiene Science, Gangneung-Wonju National University, Gangneung, Republic of Korea. Conceptualisation, reviewed and edited the manuscript, read and approved the final version of the manuscript.; c Professor, Department of Dental Hygiene, College of Dentistry; Research Institute of Oral Science; Research Institute of Dental Hygiene Science, Gangneung-Wonju National University, Gangneung, Republic of Korea. Conceptualisation, reviewed and edited the manuscript, read and approved the final version of the manuscript.; d Professor, Department of Dental Hygiene, College of Dentistry; Research Institute of Oral Science; Research Institute of Dental Hygiene Science, Gangneung-Wonju National University, Gangneung, Republic of Korea. Conceptualisation, methodology, prepared original draft, read and approved the final version of the manuscript.

**Keywords:** periodontal disease, periodontitis, sleep, sleep duration, vitamin D, vitamin D deficiency

## Abstract

**Purpose:**

This study aimed to investigate the association between vitamin D levels and periodontitis according to sleep duration in a representative sample of Korean adults.

**Materials and Methods:**

A total of 3535 subjects who participated in the sixth (2013–2014) Korea National Health and Nutrition Examination Survey were examined. Vitamin D deficiency was defined as a 25-hydroxyvitamin D serum concentration of < 20 ng/ml. Periodontal status was assessed with the community periodontal index (CPI). A high CPI was defined as a score ≥ 3. Multivariable logistic regression analyses were adjusted for sociodemographic variables, oral and general health behaviors, and systemic health status. All analyses used a complex sampling design, and a subgroup analysis was performed to determine estimates following stratification for sleep duration (≤ 5, 6, 7–8, and ≥ 9 h per day).

**Results:**

Multivariable regression analysis indicated that among participants who slept for ≥ 9 h per day, those with vitamin D deficiency were 5.51 times (95% confidence interval = 2.04–14.89) more likely to have periodontitis than those with sufficient vitamin D levels. This association was not statistically significant in the other sleep duration groups.

**Conclusion:**

The findings of this study indicate that people with vitamin D deficiency who sleep 9 h or longer may also be statistically significantly more likely to have periodontitis.

Vitamin D is a fat-soluble vitamin that can be synthesised in the human skin when exposed to adequate sunlight, unlike other essential vitamins that require external intake through dietary means.^19^ Vitamin D is necessary for regulating calcium and phosphorus metabolism, as well as parathyroid hormone levels,^3^ thereby maintaining bone health.^1^ As it can modulate inflammatory responses, vitamin D has a protective effect against conditions such as cardiovascular disease, hypertension, osteoporosis, diabetes, and cancer.^17,19^

Vitamin D deficiency has been estimated to affect approximately one billion people worldwide.^21^ According to the Korea National Health and Nutrition Examination Survey (KNHANES) in 2014, the prevalence of vitamin D deficiency (≤ 20 ng/ml) among adults aged 19 years and older in Korea was 75.5% in males and 83.2% in females (results from raw data analysis). Vitamin D deficiency is primarily caused by the lack of sunlight exposure or a major source of vitamin D, which may be associated with obesity, liver failure, pregnancy, and aging.^20^

Previous studies have suggested that vitamin D deficiency affects periodontal health.^6,11,30^ Low vitamin D levels in US adults were associated with periodontitis, independent of bone mineral density (BMD).^9^ As vitamin D has immunomodulatory effects as well as effects on BMD,^9,28^ it might affect the development of periodontitis. Several previous studies have reported an association between polymorphisms in the vitamin D receptor gene and periodontitis through their effects on immune regulation and bone metabolism.^6,39^

Sleep habits may have a significant impact on the immune system and the risk of many systemic diseases.^24,25^ Sleep duration, sleep quality, and sleep disorders can affect the systemic inflammatory status and oxidative stress, thereby increasing the risk of developing many systemic diseases.^7,34,35^ Recent studies have evaluated the effects of sleep duration on vitamin D and periodontal status. Their results suggest that the relationship between sleep duration and vitamin D deficiency may be bidirectional.^8,33^ Furthermore, sleep disorders may lead to an unhealthy periodontal status.^14,29^ Han et al^15^ reported that extra-long (> 9 h) periods of sleep and sleeping during daytime hours were associated with periodontitis.

Taken together, it can be hypothesised that the association between vitamin D deficiency and periodontal conditions may vary according to sleep duration, which is an important factor in the maintenance of systemic health. However, to date, very few studies have investigated this potential relationship. Therefore, this study aimed to evaluated the association between vitamin D deficiency and periodontitis according to sleep duration.

## MATERIALS AND METHODS

### Data Source and Study Population

In this cross-sectional study, the data included a subset of the KNHANES conducted by the Korea Disease Control and Prevention Agency (KDCA) from 2013 to 2014 and data acquired in the first and second years of the sixth KNHANES (VI). The KNHANES was conducted after obtaining approval from the Institutional Review Board of the National Statistical Office and KDCA (first year: 2013-07CON-03-4C; second year: 2013-12EXP-03-5C). All survey subjects participated voluntarily and provided informed consent.

The sampling protocol of the KNHANES comprised a complex, stratified, multistage, and probability cluster survey of a representative sample of the civilian population in Korea. The population of the survey included all non-institutionalised civilians aged ≥ 1 year. The survey used stratified multistage probability sampling units based on geographic area, age, and gender, which were determined via the most recent 5-year national census in Korea (2010 National Census Registry). Using the 2010 data, 192 primary sampling units were selected across Korea. The final set for the KNHANES included 3840 households. 14,930 participants aged 19 years provided data on one or more variables from 2013 to 2014. Among these participants, 3535 were evaluated for blood vitamin D levels and periodontal conditions. Written informed consent was obtained from all participants.

### Serum Vitamin D Levels

Vitamin D levels were assessed using whole blood levels; a 1-ml sample of blood was obtained from each participant for radioimmunoassay. Vitamin D from the diet and skin is metabolised in the liver to 25-hydroxyvitamin D (25(OH)D), which is used to determine a subject’s vitamin D level.^18^ Vitamin D deficiency was defined as a serum 25(OH)D concentration of ≤ 20 ng/ml. This cutoff point followed the suggestions of the National Academy of Medicine (known as the Institute of Medicine) and the Pediatric Endocrine Society and was equivalent to the standards used in many laboratories.^12^

### Periodontal Conditions

The community periodontal index (CPI) was used to assess the periodontal conditions. A high CPI was defined as a score ≥ 3. A CPI of 3 shows that at least one index tooth site has a probing pocket depth (PD) greater than 3.5 mm; a CPI of 4 indicates a PD of 5.5 mm or greater. A specially designed CPI probe was used according to World Health Organization (WHO) guidelines.^40^ Approximately 20 g of probing force was used. The probe tip was gently inserted at each site, and the total extent of the sulcus or pocket was explored. Twenty-one trained dentists examined the periodontal conditions of the participants in the sixth KNHANES. The inter-examiner mean Kappa value was 0.84 (0.60 to 0.88) during the first half of the year and 0.86 (0.66 to 0.91) during the second half of the year.

### Covariates

Covariates were classified into three groups according to the characteristics. The first group of sociodemographic variables comprised the following: age, gender, educational level, and household income. Educational levels were assessed based on the highest diploma achieved. Household income referred to the monthly average family income adjusted for the number of family members and categorised into quartiles. The second group included oral and general health behaviours, such as floss use, interproximal toothbrush use, monthly alcohol consumption, and current smoking status. The third group comprised variables related to general health such as diabetes and obesity. Sociodemographic variables, oral and general health behaviours, and systemic health status were examined using a self-administered questionnaire survey.

### Statistical Analysis

Individual weighted factors were used and the complex sampling design of the survey was used to obtain variances. Multivariable logistic regression analysis was conducted to examine the association between vitamin D deficiency and CPI, adjusted for the effects of covariates in the logistic model (age, gender, educational level, household income, use of floss and interproximal toothbrush, monthly alcohol consumption, current smoking, diabetes, and obesity). Subgroup analysis was conducted to determine the estimates stratified according to sleep duration, which was a confounding factor. Sleep duration was assessed through a self-administered survey, and responses to the question of mean number of hours of sleep per day were categorised for stratification analysis. The participants were divided into four groups based on sleep duration (≤ 5, 6, 7–8, and ≥ 9 h per day) for the subgroup analysis, considering the WHO’s recommended time and previous studies.^15^ All statistical analyses were conducted using SPSS version 23.0 software (IBM; Armonk, NY, USA).

## RESULTS

Table 1 shows the characteristics of the study participants categorised according to their periodontal condition. The mean age was 37.43 ± 0.27 years in the low CPI group and 50.14 ± 0.42 years in the high CPI group. The percentage of participants with a high CPI was 23.6% in the vitamin D-deficient group, and the crude OR of vitamin D deficiency for a high CPI was 0.66 (95% CI: 0.54 to 0.80). The percentages of participants with a high CPI were 31.3% and 22.6% among those who slept for ≤ 5 and ≥ 9 h, respectively.

**Table 1:** Univariate analysis of sociodemographic, oral health behaviours, and general health variables based on CPI

Variables	Low CPI		High CPI		OR (95% CI)^b^
	n	% (SE)^a^	n	% (SE)^a^	
Age (n = 3690)	37.43 ± 0.27^c^		50.14 ± 0.42^c^		1.07 (1.07 – 1.08)
**Sex (n = 3690)**					
Male	1178	69.7 (1.3)	619	30.3 (1.3)	1.81 (1.55 – 2.11)
Female	1478	80.6 (1.1)	415	19.4 (1.1)	reference
**Highest diploma (n = 3466)**					
Primary school	243	47.3 (2.9)	262	52.7 (2.9)	5.22 (4.00 – 6.82)
Middle school	202	62.6 (3.1)	131	37.4 (3.1)	2.80 (2.06 – 3.81)
High school	1030	76.8 (1.4)	342	23.2 (1.4)	1.42 (1.16 – 1.74)
≥ University or college	1028	82.4 (1.2)	228	17.6 (1.2)	reference
**Household income^d^ (n=3675)**					
< 25%	289	64.6 (2.7)	192	35.4 (2.7)	1.88 (1.41 – 2.50)
25 – 50%	696	73.3 (1.6)	304	26.7 (1.6)	1.25 (1.00 – 1.57)
50 – 75%	813	75.7 (1.6)	273	24.3 (1.6)	1.10 (0.87 – 1.39)
> 75%	846	77.4 (1.6)	262	22.6 (1.6)	reference
**Use of floss (n = 3529)**					
No	1908	71.5 (1.2)	862	28.5 (1.2)	2.46 (1.97 – 3.07)
Yes	638	86.1 (1.3)	121	13.9 (1.3)	reference
**Use of interproximal tooth brush (n = 3529)**					
No	1983	74.7 (1.1)	771	25.3 (1.1)	1.00 (0.80 – 1.25)
Yes	563	74.7 (2.0)	212	25.3 (2.0)	reference
**Current smoker (n = 3532)**					
No	2049	78.3 (1.1)	672	21.7 (1.1)	reference
Yes	501	64.9 (2.0)	310	35.1 (2.0)	1.96 (1.61 – 2.38)
**Monthly alcohol consumption (n = 3535)**					
No	1018	72.9 (1.5)	424	27.1 (1.5)	reference
Yes	1533	75.7 (1.2)	560	24.3 (1.2)	0.86 (0.72 – 1.03)
**Vitamin D deficiency (n = 3690)^e^**					
Yes (< 20 ng/ml)	2046	76.4 (1.1)	699	23.6 (1.1)	0.66 (0.54 – 0.80)
No (≥ 20 ng/ml)	610	67.9 (2.0)	335	32.1 (2.0)	reference
**Diabetes/glucose status (n = 3426)**					
Normal	1895	80.6 (1.1)	536	19.4 (1.1)	reference
Impaired fasting glucose	451	63.5 (2.2)	265	36.5 (2.2)	2.39 (1.94 – 2.94)
Diabetes	128	44.9 (3.4)	151	55.1 (3.4)	5.10 (3.77 – 6.89)
**Obesity/body weight status (n = 3675)**					
Underweight	139	87.1 (2.9)	23	12.9 (2.9)	0.49 (0.30 – 0.82)
Normal	1735	77.0 (1.2)	583	23.0 (1.2)	reference
Obese	767	67.2 (1.6)	428	32.8 (1.6)	1.64 (1.37 – 1.95)
**Sleep duration (n = 3535)**					
≤5 h	347	68.7 (2.7)	165	31.3 (2.7)	1.41 (1.08 – 1.84)
6 h	716	75.2 (1.7)	263	24.8 (1.7)	1.02 (0.84 – 1.24)
7–8 h	1326	75.6 (1.2)	488	24.4 (1.2)	reference
≥9 h	162	77.4 (3.0)	68	22.6 (3.0)	0.91 (0.64 – 1.28)

^a^Weighted percentage ± standard error. ^b^Crude odds ratio and 95% confidence interval for higher CPI. ^c^Weighted mean and standard error. ^d^Household income: monthly average family equivalent income (= monthly average household income/√(the number of household members). ^e^Vitamin D deficiency was defined as a serum 25(OH)D level of 20 ng/ml. CPI, community periodontal index; OR, odds ratio.

The characteristics of the study participants, categorised by sleep duration, are shown in Table 2. Among participants who had vitamin D deficiency, 13.0% slept for ≤ 5 h and 7.2% slept for ≥ 9 h. Among participants who had a high CPI, 16.8% slept for ≤ 5 h; this was greater than the percentage of participants (12.5%) who slept for the same duration and had a low CPI. Among participants who had a high CPI, 6.3% slept for ≥ 9 h; this was lower than the percentage of participants (7.3%) who slept for the same duration and had a low CPI.

**Table 2:** Univariate analysis of sociodemographic and oral health variables, and general health variables based on sleep duration

Variables	Sleep duration							
	≤ 5 h		6 h		7–8 h		≥ 9 h	
	n	% (SE)^a^	n	% (SE)^a^	n	% (SE)^a^	n	% (SE)^a^
Age (n = 3535)	44.62 ± 0.72^b^		41.14 ± 0.48^b^		40.03 ± 0.36^b^		35.03 ± 1.13^b^	
**Gender (n = 3535)**								
Male	225	13.3 (0.9)	486	28.4 (1.1)	898	52.5 (1.3)	93	5.8 (0.7)
Female	287	14.0 (0.8)	493	26.5 (1.1)	916	50.9 (1.3)	137	8.6 (0.8)
**Highest diploma (n = 3456)**								
Primary school	135	23.3 (2.1)	123	22.6 (1.9)	206	45.5 (2.7)	38	8.5 (1.5)
Middle school	73	22.4 (2.5)	88	27.5 (2.7)	144	42.5 (3.0)	26	7.5 (1.7)
High school	167	12.8 (1.0)	387	26.8 (1.3)	711	51.7 (1.5)	102	8.7 (0.9)
≥ University or college	130	10.4 (1.0)	355	29.3 (1.3)	707	55.2 (1.5)	64	5.1 (0.7)
**Household income^c^ (n = 3525)**								
< 25%	101	18.4 (2.0)	125	26.3 (2.5)	191	43.4 (2.8)	40	11.9 (2.0)
25 – 50%	141	13.1 (1.2)	250	25.7 (1.6)	497	52.9 (1.8)	69	8.4 (1.2)
50 – 75%	147	14.4 (1.3)	293	27.9 (1.5)	533	50.8 (1.7)	74	6.8 (0.9)
> 75%	122	11.6 (1.2)	308	29.2 (1.5)	589	55.0 (1.6)	45	4.3 (0.7)
**Use of floss (n = 3529)**								
No	416	14.1 (0.7)	769	27.6 (0.9)	1403	51.2 (1.0)	182	7.1 (0.6)
Yes	96	12.0 (1.3)	209	27.3 (1.8)	406	53.9 (2.0)	48	6.8 (1.1)
**Use of interproximal tooth brush (n = 3529)**								
No	398	13.2 (0.7)	759	27.4 (0.9)	1417	52.0 (1.1)	180	7.4 (0.6)
Yes	114	15.0 (1.6)	219	28.2 (1.7)	392	51.0 (1.9)	50	5.8 (0.9)
**Current smoker (n = 3532)**								
No	383	12.6 (0.7)	752	27.3 (1.0)	1410	53.0 (1.1)	176	7.1 (0.6)
Yes	129	16.4 (1.4)	226	28.1 (1.9)	402	48.6 (2.1)	54	6.9 (1.1)
**Monthly alcohol consumption (n = 3535)**								
No	233	14.4 (1.0)	374	25.8 (1.3)	741	52.5 (1.4)	94	7.3 (0.9)
Yes	279	13.1 (0.9)	605	28.6 (1.1)	1073	51.4 (1.2)	136	6.9 (0.6)
**Vitamin D deficiency (n = 3535)^d^**								
Yes (< 20 ng/ml)	367	13.0 (0.8)	731	27.2 (0.9)	1362	52.6 (1.0)	172	7.2 (0.6)
No (≥ 20 ng/ml)	145	15.5 (1.4)	248	28.9 (1.7)	452	49.1 (1.8)	58	6.5 (0.9)
**Diabetes/glucose status (n = 3413)**								
Normal	328	12.4 (0.8)	667	27.2 (1.0)	1267	52.9 (1.1)	161	7.5 (0.7)
Impaired fasting glucose	112	15.7 (1.5)	211	29.4 (1.9)	352	49.7 (2.0)	39	5.2 (0.9)
Diabetes	53	18.3 (2.5)	65	26.0 (3.0)	131	45.3 (3.4)	27	10.4 (2.2)
**Obesity/body weight status (n = 3522)**								
Underweight	15	6.9 (2.0)	40	23.8 (4.0)	78	49.1 (4.5)	27	20.2 (4.0)
Normal	315	13.1 (0.8)	608	26.8 (1.0)	1174	53.6 (1.1)	130	6.5 (0.6)
Obese	182	15.8 (1.3)	327	29.6 (1.5)	554	48.6 (1.7)	72	6.0 (0.8)
**Periodontal status (n = 3535)**								
Low CPI	347	12.5 (0.7)	716	27.7 (0.9)	1326	52.4 (1.1)	162	7.3 (0.6)
High CPI	165	16.8 (1.5)	263	27.0 (1.6)	488	49.9 (1.8)	68	6.3 (0.9)

^a^Weighted percentage ± standard error. ^b^Crude odds ratio and 95% confidence interval for higher CPI. ^c^Household income: monthly average family equivalent income (= monthly average household income/√(the number of household members). ^d^Vitamin D deficiency was defined as a serum 25(OH)D level of 20 ng/ml. CPI, community periodontal index; SE, standard error.

The results of the subgroup analyses for sleep duration as a statistically significant confounding factor are presented in Table 3. The association between CPI and vitamin D deficiency differed according to the sleep duration strata. Among participants who slept ≥ 9 h, those with vitamin D deficiency were 5.51 (95% CI: 2.04 to 14.89) times more likely to have a high CPI than participants without vitamin D deficiency. Vitamin D levels were not associated with a high CPI in the other sleep duration groups, including those who slept for ≤ 5 h.

**Table 3:** Association of CPI with vitamin D deficiency^a^ according to sleep duration

Groups	Age-adjusted model		Multivariable model	
	Adjusted OR	95% CI	Adjusted OR	95% CI
Total	0.99	0.80 – 1.23	1.09	0.86 – 1.38
≤5 h	1.30	0.79 – 2.15	1.68	0.98 – 2.87
6 h	0.79	0.55 – 1.15	0.83	0.55 – 1.27
7–8 h	0.92	0.68 – 1.25	0.97	0.70 – 1.34
≥9 h	3.69	1.51 – 9.04	5.51	2.04 – 14.89

^a^Vitamin D deficiency was defined as a serum 25-hydroxyvitamin D level of 20 ng/ml. The multivariable logistic regression model was adjusted for sociodemographic variables (age, gender, educational level, and household income), oral health behaviors (use of floss and inter-proximal toothbrush), health behaviours (monthly alcohol consumption and current smoking), and systemic health status (diabetes and obesity). CPI, community periodontal index.

## DISCUSSION

In this study, the association between poor periodontal health and vitamin D deficiency, as indicated by a high CPI, was only statistically significant among participants who slept for ≥ 9 h per day. This association was observed after adjusting for socioeconomic variables, oral and general health behaviours, and general health status.

Several studies have reported a relationship between vitamin D levels and periodontal health.^6,9,11,30^ As vitamin D levels are regulated by bone metabolism, which involves calcium homeostasis and anti-inflammatory effects, deficiencies may be associated with an increased risk of periodontitis. In addition, vitamin D receptor gene polymorphisms could have an influence on the immune system and bone metabolism.^39^

This study confirmed the results of the previous study described above, but only found an association between vitamin D deficiency and high CPI in participants who overslept. Previous epidemiological studies have suggested that both short and long sleep durations may be associated with poor periodontal health and adverse health effects, such as diabetes, cardiovascular disease, and obesity.^16,27,38^ Sleep habits are a very important factor throughout the course of life, as they affect systemic inflammatory responses and the immune system, and are essential for the natural circadian rhythm.^23^ Several studies have shown that sleep disorders (e.g., short or long sleep duration, sleep apnea, and poor sleep quality) may alter hormonal and cytokine levels, thereby increasing the risk of systemic disease.^22^ Too little or too much sleep can affect hormones and inflammatory processes, resulting in inflammatory responses by the immune system, which can increase the risk of various systemic diseases. Thus, systemic health status may vary depending on sleep duration.

In this study, sleep duration was a confounding factor affecting the association between vitamin D deficiency and poor periodontal health. Stratified analyses revealed a statistically significant association between vitamin D deficiency and high CPI in participants who slept for ≥ 9 h per day. Therefore, long sleep duration may be a key factor in the association between vitamin D deficiency and periodontal conditions. These results may be attributed to the impact of long sleep duration, which is associated with systemic inflammation and immune system impairment, on vitamin D deficiency-induced systemic inflammation and decreases in immunity. It is associated with periodontitis through the interaction of bacterial, host, and environmental factors.^36^ McCarty and Marino^32^ suggested that lower levels of 25(OH)D negatively affect sleep by activating proinflammatory mediators. In particular, 25(OH)D (a vitamin D metabolite) is usually stored in skeletal muscle cells, and muscle function can be influenced by sleep duration.^5,31^ If the sleep requirements for adequate muscle function are not fulfilled, vitamin D deficiency may develop. Thus, long sleep durations may affect vitamin D levels and their association with periodontitis. Moreover, vitamin D plays a significant role in bone health,^2^ and sleep is a key factor in bone metabolism.^37^ A long sleep duration may lead to poor bone metabolism due to vitamin D deficiency, which could in turn induce periodontal tissue destruction. Our findings may also be explained by oral and general health variables (e.g., low socioeconomic status, poor general health, and depression) that are related to longer sleep durations and periodontitis.^13^

Some limitations in this study are acknowledged. First, it was not possible to identify causality in the relationship between periodontitis and vitamin D deficiency, due to the cross-sectional study design. Second, periodontal conditions were assessed using the CPI. Therefore, periodontal diseases could have been over- or underestimated due to the presence of pseudo-pockets and the use of index teeth.^26^ While the diagnosis of periodontal disease is usually based on the severity and extent of clinical attachment loss and PD,^10^ the CPI is an epidemiological tool developed by the WHO for the evaluation of periodontal status in population surveys.^4^ Third, inflammatory markers and immunity-related variables were not included in the logistic regression models. These variables could help further the understanding of the mechanisms by which sleep duration influences the association between vitamin D and periodontal status and warrant consideration in future studies. Thus, the results of this study should be interpreted with caution. In this study, we analysed the association between vitamin D levels and periodontal health according to sleep duration through multivariable regression models, considering sociodemographic variables, oral and general health behaviours, and systemic health status as potential confounding factors. Despite adjusting for the confounding variables that may affect this association, multicollinearity and the potential for omitted confounding variables should be taken into account. Nevertheless, to the best of our knowledge, no prior studies using nationally representative data from Korean adults have evaluated sleep duration as a confounding factor in the association between vitamin D deficiency and periodontal status. This is pertinent, as the relationship between vitamin D deficiency and periodontal status may vary greatly if key confounding factors such as sleep duration are not accounted for.

## CONCLUSIONS

People with vitamin D deficiency were statistically significantly more likely to have periodontitis if they slept ≥ 9 h per day. Our results suggest that sleep duration, vitamin D levels, and periodontal health should be controlled and managed to maintain oral and general health throughout life.
